# Response and survival of breast cancer intrinsic subtypes following multi-agent neoadjuvant chemotherapy

**DOI:** 10.1186/s12916-015-0540-z

**Published:** 2015-12-18

**Authors:** Aleix Prat, Cheng Fan, Aranzazu Fernández, Katherine A. Hoadley, Rossella Martinello, Maria Vidal, Margarita Viladot, Estela Pineda, Ana Arance, Montserrat Muñoz, Laia Paré, Maggie C. U. Cheang, Barbara Adamo, Charles M. Perou

**Affiliations:** Translational Genomics Group, Vall d’Hebron Institute of Oncology (VHIO), Barcelona, Spain; Translational Genomics and Targeted Therapeutics in Solid Tumors, August Pi i Sunyer Biomedical Research Institute (IDIBAPS), Rosselló, 149, 08036 Barcelona, Spain; Department of Medical Oncology, Hospital Clínic de Barcelona, Barcelona, Spain; Lineberger Comprehensive Cancer Center, University of North Carolina, Chapel Hill, NC USA; Clinical Trials & Statistics Unit, The Institute of Cancer Research, Belmont, UK; Department of Genetics, University of North Carolina, Chapel Hill, NC USA; Department of Pathology & Laboratory Medicine, University of North Carolina, Chapel Hill, NC USA

**Keywords:** Biomarker, Breast cancer, Gene expression, Intrinsic subtypes, Triple-negative

## Abstract

**Background:**

Predicting treatment benefit and/or outcome before any therapeutic intervention has taken place would be clinically very useful. Herein, we evaluate the ability of the intrinsic subtypes and the risk of relapse score at diagnosis to predict survival and response following neoadjuvant chemotherapy. In addition, we evaluated the ability of the Claudin-low and 7-TNBCtype classifications to predict response within triple-negative breast cancer (TNBC).

**Methods:**

Gene expression and clinical-pathological data were evaluated in a combined dataset of 957 breast cancer patients, including 350 with TNBC, treated with sequential anthracycline and anti-microtubule-based neoadjuvant regimens. Intrinsic subtype, risk of relapse score based on subtype and proliferation (ROR-P), the Claudin-low subtype and the 7-TNBCtype subtype classification were evaluated. Logistic regression models for pathological complete response (pCR) and Cox models for distant relapse-free survival (DRFS) were used.

**Results:**

Basal-like, Luminal A, Luminal B, and HER2-enriched subtypes represented 32.7 %, 30.6 %, 18.2 %, and 10.3 % of cases, respectively. Intrinsic subtype was independently associated with pCR in all patients, in hormone receptor-positive/HER2-negative disease, in HER2-positive disease, and in TNBC. The pCR rate of Basal-like disease was >35 % across all clinical cohorts. Neither the Claudin-low nor the 7-TNBCtype subtype classifications predicted pCR within TNBCs after accounting for intrinsic subtype. Finally, intrinsic subtype and ROR-P provided independent prognostic information beyond clinicopathological variables and type of pathological response. A 5-year DRFS of 97.5 % (92.8–100.0 %) was observed in these neoadjuvant-treated and clinically node-negative patients predicted to be low risk by ROR-P (i.e. 57.4 % of Luminal A tumors with clinically node-negative disease).

**Conclusions:**

Intrinsic subtyping at diagnosis provides prognostic and predictive information for patients receiving neoadjuvant chemotherapy. Although we could not exclude a survival benefit of neoadjuvant chemotherapy in patients with early breast cancer with clinically node-negative and ROR-low disease at diagnosis, the absolute benefit of cytotoxic therapy in this group might be rather small (if any).

**Electronic supplementary material:**

The online version of this article (doi:10.1186/s12916-015-0540-z) contains supplementary material, which is available to authorized users.

## Background

During the last decade, it has become apparent that gene expression-based data in breast cancer can provide useful biological, prognostic, and predictive information [1, 2]. For example, the main intrinsic molecular subtypes of breast cancer (Luminal A, Luminal B, HER2-enriched, and Basal-like) are biologically and prognostically relevant [3–6] and have been associated with anthracycline and tamoxifen benefit in the adjuvant setting [7–9]. Importantly, the intrinsic subtypes are not fully recapitulated by the combined determination of pathology-based biomarkers such as estrogen receptor (ER), progesterone receptor (PR), Ki67, and HER2 [1, 3, 4, 9–12], all of which are currently being used in the clinical setting. Thus, from a clinical perspective, there is a need to understand the value of identifying the intrinsic subtypes, as well as other gene expression-based classifications, beyond clinicopathological variables.

We have previously shown that all the intrinsic subtypes can be identified within various clinically-defined groups, albeit with different proportions [9, 11, 13, 14]. For example, although the Basal-like subtype predominates within triple-negative breast cancer (TNBC), all the intrinsic subtypes can be identified in TNBC, and identification of the ‘Basal-like versus not’ classification within TNBC might be clinically relevant [15, 16]. Beyond the main subtypes of breast cancer, we have also reported the Claudin-low subtype characterized by the low to absent expression of luminal differentiation markers, and by the high enrichment for epithelial-to-mesenchymal transition markers, immune response genes, and cancer stem cell-like features [4]. In a previous report, Claudin-low tumors showed an intermediate pathological complete response (pCR) rate compared to Basal-like tumors in a cohort of 133 patients with TNBC and non-TNBC tumors treated with anthracycline/taxane-based chemotherapy [4].

Recently, Lehmann et al. [17] reported the identification of seven different potential molecular subtypes of TNBC (Basal 1 (BL1), Basal 2 (BL2), Immunomodulatory, Luminal androgen receptor (LAR), Mesenchymal, Mesenchymal stem cell (MSL), and unstable UNS). This seven-subtype classification of TNBC was found to be associated with pCR in an independent cohort of 130 TNBC patients treated with anthracycline/taxane-based chemotherapy [18]. Among the different subtypes, BL2 and LAR subtypes showed the lowest pCR rates, and BL1 showed the highest pCR rates, compared to the other subtypes [18].

In this study, we evaluated the ability of the common PAM50 intrinsic subtypes, and the risk of relapse score based on subtype and proliferation (ROR-P), to predict response and survival outcomes beyond standard clinical-pathological variables following neoadjuvant multi-agent chemotherapy. In addition, we evaluated the ability of the Claudin-low [4] and the seven TNBC subtype classifications [17] to predict pCR within TNBC. Finally, we trained and tested gene expression-based models predictive of pCR in all patients, in patients with Basal-like disease, and in patients with Luminal disease, to identify some of the driving biological features behind response within these groups.

## Methods

### Patients, samples and clinical data

Four clinically annotated microarray-based breast cancer datasets were evaluated from the public domain (GSE25066 [19], GSE32646 [20], GSE41998 [21], and GSE22226 [22]). All patients received sequential anthracycline and taxane/exabepilone-based neoadjuvant regimens. Patients that received trastuzumab were excluded. All gene expression microarray-based analyses were performed in pre-treatment tumor samples. The total number of patients included in this analysis was 957 (Additional file 1: Figure S1). Ethical approval and informed consent were not required for this study.

The Hatzis et al. [19] dataset includes 508 patients treated with sequential anthracycline and taxane-based chemotherapy in various research protocols: LAB99-402, USO-02-103, 2003-0321, and I-SPY-1. A total of 508 patients from the Hatzis et al. [19] dataset have follow-up data. Patients with any nuclear immunostaining of ER in the tumor cells were considered eligible for adjuvant endocrine therapy. In Horak et al. [21], 279 patients were randomized to four cycles of doxorubicin/cyclophosphamide followed by 1:1 randomization to either ixabepilone 40 mg/m^2^ every 3 weeks for four cycles or weekly paclitaxel 80 mg/m^2^ for 12 weeks, followed by either weekly paclitaxel or exabepilone for 3 months. In Miyake et al. [20], 115 patients received paclitaxel (80 mg/m^2^) weekly for 12 cycles followed by 5-FU (500 mg/m^2^), epirubicin (75 mg/m^2^) and cyclophosphamide (500 mg/m^2^) every 3 weeks for four cycles. Finally, Essermann et al. [22] included 149 patients treated in the ISPY-1 clinical trial with doxorubicin/cyclophosphamide followed by paclitaxel. In this dataset, we excluded 80 patients that were already included in Hatzis et al. [19], one patient that received doxorubicin/cyclophosphamide-only, and 13 patients that received trastuzumab.

### Pathology-based subtype definitions

We used the pathological ER, PR, and HER2 statuses of each tumor sample as provided in each dataset [19–22]. The following pathology-based subtype definitions were evaluated: hormone receptor (HR)^+^/HER2^–^, HER2^+^, and TNBC.

### Pathological complete response (pCR) definition

pCR across all cohorts was defined as the percentage of patients with no histologic evidence of residual invasive carcinoma in the breast and axillary lymph nodes, regardless of the presence or absence of ductal carcinoma in situ.

### Identification of the intrinsic subtypes

In each dataset, all tumors were assigned to an intrinsic molecular subtypes of breast cancer (Luminal A, Luminal B, HER2-enriched, Basal-like) and the Normal breast-like group using the PAM50 subtype predictor as previously described [4, 22–24]. For the ISPY-1 [22] and Miyake [20] cohorts, we used the previously reported subtype calls [22, 25]. In addition, we evaluated the previously reported ROR-P score [23]. To identify the Claudin-low subtype [4] in TNBC, we applied the nine cell-line Claudin-low predictor in each microarray dataset using all patients as previously described [4]. TNBCs that were identified as Claudin-low were considered Claudin-low regardless of the intrinsic subtype call.

### Identification of subtypes within TNBC

To identify the seven TNBC subtypes described by Lehmann et al. [17], we first selected the TNBCs from each dataset. Secondly, we submitted the raw data of each individual dataset to the TNBCtype online predictor (http://cbc.mc.vanderbilt.edu/tnbc/) [26]. The TNBCtype tool first checks the levels of the ER gene (ESR1) across all TNBCs, and identifies those samples with a relative high ESR1 expression level. These ESR-high TNBCs need to be removed from each dataset in order for the TNBCtype predictor algorithm to continue.

### Training and testing gene expression-based models

We explored the ability of newly derived gene expression-based models to predict pCR in three different cohorts: all patients, patients with Basal-like disease, and patients with Luminal disease (Luminal A and B combined). To build each model, we explored the expression of 378 different gene signatures (Additional file 2: Supplemental Data) and used Elastic Net building model by 10 cross-validations. To accomplish this, we used the MDACC-based cohort (GSE25066 [19]) as a training set where each model was derived in each cohort, and then tested this exact model in the same clinical cohorts on the other datasets (testing sets). To estimate the performance of each model, we used the area under the receiver operating characteristic (auROC) curves.

### Statistical analysis

Biologic analysis of the gene list was performed with DAVID annotation tool (http://david.abcc.ncifcrf.gov/) [27]. Association between subtype and pCR was assessed by univariate and multivariable logistic regression analysis. Likelihood ratio tests were used to assess if a variable added predictive information to each model. To estimate the predictive performance of each variable, auROC curves were evaluated. Survival functions to distant relapse-free survival (DRFS) were from the Kaplan-Meier product-limit estimator with tests of differences by the log-rank test. Cox proportional hazard models adjusted for standard clinical-pathological variables were used to test the independent associations with survival of each variable. Reported *P* values are two-sided.

## Results

### Clinical-pathological characteristics of the combined cohort

A total of 957 patients with breast cancer treated with sequential anthracycline and taxane/ixabepilone-based neoadjuvant regimens were included in the analysis (Table 1). All datasets included all clinicopathological variables, except for histological grade and nodal status in Horak et al. [19] and nodal status in ISPY-1 et al. [22] since these were not provided. The mean age was 50.0 years and most patients had tumors of less than 5 cm (61.3 % T0-T2) and positive axillary nodal status by clinical assessment (69.7 %). Pathology-based subtype distribution was as follows: 494 (52.7 %) HR^+^/HER2^–^, 93 (9.9 %) HER2^+^, and 350 (37.4 %) TNBCs.

**Table 1:** Clinicopathological characteristics and subtype distribution of the combined cohort evaluated in this study

	GSE41998 [21]	%	GSE25066 [19]	%	GSE32646 [20]	%	GSE22226 [22]	%	TOTAL	%
N	279	–	508	–	115	–	55	–	957	–
Age, years (mean)	49	–	50	–	51	–	49	–	50	–
Tumor size										
T0-T2	177	64.1 %	288	56.7 %	92	80 %	27	50.0 %	584	61.3 %
T3-T4	99	35.9 %	220	43.3 %	23	20 %	27	50.0 %	369	38.7 %
ER IHC status										
Positive	108	38.7 %	297	59.2 %	71	62 %	31	58.5 %	507	53.4 %
Negative	171	61.3 %	205	40.8 %	44	38 %	22	41.5 %	442	46.6 %
PR IHC status										
Positive	99	35.6 %	243	48.5 %	45	39 %	21	39.6 %	408	43.1 %
Negative	179	64.4 %	258	51.5 %	70	61 %	32	60.4 %	539	56.9 %
Triple-negative status										
No	139	49.8 %	330	65.0 %	89	77 %	47	88.7 %	605	63.4 %
Yes	140	50.2 %	178	35.0 %	26	23 %	6	11.3 %	350	36.6 %
HER2 IHC/FISH status										
Negative	251	90.0 %	485	98.8 %	81	70 %	24	49.0 %	841	90.0 %
Positive	28	10.0 %	6	1.2 %	34	30 %	25	51.0 %	93	10.0 %
Histological grade										
1	–	–	32	6.8 %	16	13.9 %	2	3.6 %	50	14.9 %
2	–	–	180	38.2 %	78	67.8 %	27	49.1 %	285	48.2 %
3	–	–	259	55.0 %	21	18.3 %	26	47.3 %	306	51.8 %
Nodal status										
N0	–	–	157	31 %	32	28 %	–	–	189	30.3 %
N1-3	–	–	351	69 %	83	72 %	–	–	434	69.7 %
pCR rate										
No	184	72.7 %	389	79.7 %	88	77 %	37	68.5 %	698	76.7 %
Yes	69	27.3 %	99	20.3 %	27	23 %	17	31.5 %	212	23.3 %
PAM50										
Luminal A	91	32.6 %	155	30.5 %	30	26 %	17	30.9 %	293	30.6 %
Luminal B	33	11.8 %	109	21.5 %	23	20 %	9	16.4 %	174	18.2 %
HER2-E	23	8.2 %	40	7.9 %	24	21 %	12	21.8 %	99	10.3 %
Basal-like	110	39.4 %	171	33.7 %	21	18 %	11	20.0 %	313	32.7 %
Normal-like	22	7.9 %	33	6.5 %	17	15 %	6	10.9 %	78	8.2 %

HER2-E, HER2-enriched; pCR, Pathological complete response; ER, Estrogen receptor; PR, Progesterone receptor; IHC, Immunohistochemistry

### Intrinsic subtype and ROR-P associations with survival outcome

A total of 508 patients from Hatzis et al. [19] had follow-up data (mean 2.98 years). In this dataset, both intrinsic subtype and ROR-P were found to be significantly associated with DRFS in univariate and multivariable analyses after adjustment for age, tumor size, nodal status, ER and PR status, HER2 status, histological grade, and tumor response (pCR vs. residual disease) (Additional file 1: Table S1 and S2). Of note, a 5-year DRFS rate of 90.2 % (95 % confidence interval (CI), 82.5–98.6 %) was observed in patients whose tumors were predicted to be low risk by ROR-P (Additional file 1: Figure S2A). This 5-year DRFS rate increased to 97.5 % (95 % CI, 92.78–100.0 %) in patients with ROR-P low disease that presented with clinically node-negative disease (Additional file 1: Figure S2B).

**Fig. 1:**
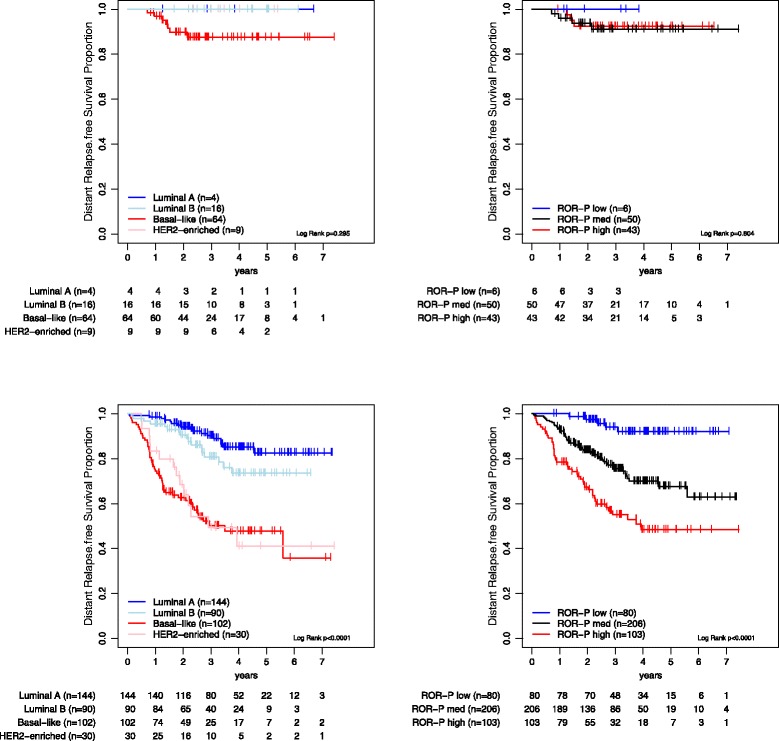
Kaplan-Meier distant relapse-free survival analysis in the MDACC-based (GSE25066 [19]) dataset based on the pathological treatment response. (**a**) Survival outcomes of the intrinsic subtypes in patients that achieved a pathological complete response (pCR); (**b**) Survival outcomes of the risk of relapse score based on subtype and proliferation (ROR-P) groups in patients that achieved a pCR; (**c**) Survival outcomes of the intrinsic subtypes in patients that did not achieve a pCR; (**d**) Survival outcomes of the ROR-P groups in patients that did not achieve a pCR

Next, we evaluated the survival outcomes based on the type of pathological response. Within patients that achieved a pCR, no variable was found to be significantly associated with DRFS in univariate analyses (Fig. 1a and b; Additional file 1: Tables S3 and S4). Within patients that did not achieve a pCR, both intrinsic subtype and ROR-P were found to be significantly associated with DRFS in univariate and multivariable analyses after adjustment for the other clinicopathological variables (Fig. 1c and d and Table 2; Additional file 1: Table S5). Among them, tumor size and nodal status before treatment were significantly associated with DRFS. Finally, high 5-year DRFS rates were observed as in the global population in patients with ROR-P low disease that did not achieve a pCR (5-year DRFS of 92.0 % (95 % CI, 85.5–99.1 %) in all patients and of 97.4 % (95 % CI, 92.6–100.0 %) in node-negative disease). No statistically significant interaction (*P* = 0.430) was observed between ROR-P (as a continuous variable) and pCR in DRFS analysis.

**Table 2:** Cox model distant relapse-free survival (DRFS) analyses in patients with residual disease from the MDACC-based cohort (GSE25066 [19])

				Univariate analysis				Multivariable analysis			
Variables	n	%	5-yr DRFS	HR	Lower 95 %	Upper 95 %	*P* value	HR	Lower 95 %	Upper 95 %	*P* value
Age, years (cont. variable)	–	–	–	1.0	0.98	1.01	0.590	0.98	0.97	1.01	0.129
Tumor size											
T0-T2	216	56 %	74 %	1.0	–	–	–	1.0	–	–	–
T3-T4	173	44 %	61 %	2.1	1.38	3.07	<0.001	1.5	1.16	1.92	0.002
Node status											
N0	126	32 %	85 %	1.0	–	–	–	1.0	–	–	–
N1-3	263	68 %	66 %	3.3	1.90	5.71	<0.001	2.9	1.62	5.37	<0.001
ER IHC											
Positive	129	34 %	78 %	1.0	–	–	–	1.0	–	–	–
Negative	255	66 %	47 %	4.0	2.69	6.02	<0.001	1.8	0.87	3.56	0.114
PR IHC											
Positive	175	46 %	79 %	1.0	–	–	–	1.0	–	–	–
Negative	208	54 %	54 %	3.3	2.15	5.05	<0.001	1.2	0.62	2.13	0.654
HER2 STATUS											
Negative	373	99 %	68 %	1.0	–	–	–	1.0	–	–	–
Positive	3	1 %	NA	1.1	0.16	8.16	0.900	0.5	0.07	3.65	0.485
Histological grade											
1	28	8 %	96 %	1.0	–	–	–	1.0	–	–	–
2	160	44 %	73 %	6.1	0.83	44.43	0.076	2.75	0.36	20.94	0.33
3	175	48 %	60 %	10.9	1.51	78.73	0.018	2.54	0.33	19.66	0.37
PAM50											
Luminal A	144	37 %	83 %	1.0	–	–	–	1.0	–	–	–
Luminal B	90	23 %	74 %	1.8	0.90	3.46	0.097	1.4	0.69	2.82	0.360
HER2-E	30	8 %	41 %	5.9	3.38	10.30	<0.001	2.7	1.15	6.41	0.023
Basal-like	102	26 %	48 %	5.3	2.63	10.84	<0.001	2.8	1.19	6.42	0.018
Normal-like	23	6 %	82 %	1.6	0.55	4.85	0.380	–	–	–	–

HER2-E, HER2-enriched; pCR, Pathological complete response; ER, Estrogen receptor; PR, Progesterone receptor; IHC, Immunohistochemistry

### Intrinsic subtype association with chemotherapy response in all patients

The pCR rates across the intrinsic molecular subtypes were 6 %, 16 %, 37 %, and 38 % for the Luminal A, Luminal B, HER2-enriched, and Basal-like subtypes, respectively. In a multivariable model, the intrinsic subtypes were independently associated with pCR after adjustment for age, tumor size, ER and PR statuses, histological grade, HER2 status, and study (Table 3 and Additional file 1: Table S6). Of note, ER and PR status by immunohistochemistry (IHC) did not provide independent predictive information once intrinsic subtype was introduced into the model.

**Table 3:** Logistic regression model analyses of chemotherapy response in the combined cohort^a^

			Univariate analysis					Multivariable analysis				
Signatures	n	pCR rate	OR	Lower 95 %	Upper 95 %	*P* value	auROC	OR	Lower 95 %	Upper 95 %	*P* value	auROC
Age, years (cont. variable)	–	–	1.0	0.97	1.00	0.027	0.547	0.99	0.97	1.01	0.178	0.744
Tumor size												
T0-T2	556	24 %	1.0	–	–	–	0.519	1.0	–	–	–	
T3-T4	353	12 %	0.8	0.55	1.02	0.071		0.6	0.50	0.84	0.001	
ER IHC												
Positive	487	11 %	1.0	–	–	–	0.679	1.0	–	–	–	
Negative	415	37 %	4.7	3.39	6.61	<0.001		1.8	0.99	3.34	0.052	
PR IHC												
Positive	393	12 %	1.0	–	–	–	0.643	1.0	–	–	–	
Negative	507	33 %	3.8	2.64	5.36	<0.001		1.1	0.65	1.89	0.716	
HER2 STATUS												
Negative	799	22 %	1.0	–	–	–	0.533	1.0	–	–	–	
Positive	88	35 %	2.1	1.30	3.30	0.002		1.3	0.64	2.51	0.492	
PAM50												
Luminal A	281	6 %	1.0	–	–	–	0.719	1.0	–	–	–	
Luminal B	168	16 %	3.0	1.57	5.64	0.001		3.3	1.72	6.45	<0.001	
HER2-E	93	37 %	8.9	4.69	17.09	<0.001		6.1	2.75	13.38	<0.001	
Basal-like	296	38 %	9.6	5.49	15.40	<0.001		6.1	2.94	12.66	<0.001	
Normal-like	72	29 %	6.4	3.16	12.96	<0.001		–	–	–	–	
STUDY												
HORAK	253	27 %	1.0	–	–	–	0.553	1.0	–	–	–	
ISPY	54	31 %	1.2	0.65	2.32	0.532		1.3	0.55	3.16	0.540	
MDACC508	488	20 %	0.7	0.48	0.97	0.032		0.9	0.58	1.33	0.526	
MIYAKE	115	23 %	0.8	0.49	1.37	0.443		1.0	0.55	1.95	0.910	

^a^OR, Odds ratio; auROC, Area under the receiver operating curve; HER2-E, HER2-enriched; pCR, Pathological complete response; ER, Estrogen receptor; PR, Progesterone receptor; IHC, Immunohistochemistry

### pCR rates of the intrinsic subtypes across pathology-defined subgroups

The intrinsic subtype classification was independently associated with pCR within HR^+^/HER2^–^, HER2^+^, and TNBC clinical subgroups (Table 4). Non-luminal (Basal-like and HER2-enriched) tumors, as a group, showed higher pCR rates than luminal (Luminal A and B) tumors in HR^+^/HER2^–^ (30.0 % vs. 8.9 %, adjusted OR = 4.20, 2.220–7.942), HER2^+^ (45.8 % vs. 14.3 %, adjusted OR = 5.22, 1.478–18.460), and TNBC (38.5 % vs. 18.5 %, adjusted OR = 2.89, 1.043–8.003) diseases. Among the different subtypes, the Basal-like subtype showed consistent pCR rates above 35 % across the three clinically-defined subgroups (36 %, 58 %, and 37 % in HR^+^/HER2^–^, HER2^+^, and TN subgroups, respectively). Finally, addition of the Claudin-low subtype to the PAM50 classification did not improve the ability to predict pCR in TNBC (Additional file 1: Table S7).

**Table 4:** Association of the intrinsic subtypes with chemotherapy response across the various pathology-based groups

	All patients		Luminal A		Luminal B		HER2-enriched		Basal-like		*P* value*
	n	pCR	n	pCR	n	pCR	n	pCR	n	pCR	
All subgroups	838	23 %	281	6 %	168	16 %	93	37 %	296	38 %	<0.001
HR^+^/HER2^–^	451	12 %	239	5 %	143	15 %	25	16 %	44	36 %	<0.001
HER2^+^	76	34 %	16	0 %	12	33 %	36	42 %	12	58 %	0.011
HR^–^/HER2^–^ (TN)	292	37 %	19	26 %	8	0 %	30	47 %	235	37 %	0.011

*Likelihood ratio tests: adjusting clinical features: age, clinical stage, clinical nodal status and study cohort. Hormone receptors status and HER2 status were also included in “all subgroups”

pCR, Pathological complete response; ER, Estrogen receptor; PR, Progesterone receptor

### TNBCtype association with chemotherapy response in TNBC

**Fig. 2:**
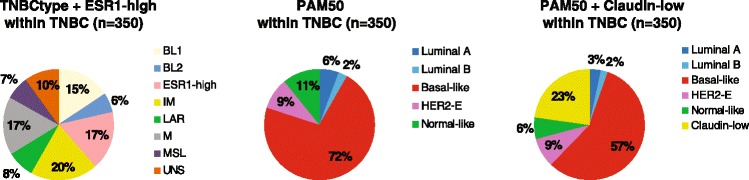
Distribution of the TNBCtype, PAM50, and PAM50 + Claudin-low subtypes within 350 clinically-defined TNBCs

Of the 350 TNBCs, 60 (17.1 %) were identified by the TNBCtype online tool [26] as having high ESR1 levels (Fig. 2) and thus were removed from many of the subsequent analyses because they are not considered a “class” by the TNBCtype tool. The intrinsic subtype distribution within this ESR1-high TNBCtype group was: Basal-like (n = 20, 33.3 %), Normal-like (n = 17, 28.3 %), Luminal A (n = 14, 23.3 %), Luminal B (n = 5, 8.3 %), and HER2-enriched (n = 4, 6.7 %). As predicted, the levels of ESR1 mRNA in the TNBCtype ESR1-high group were significantly higher than in the ESR1-low group; however, the levels of ESR1 mRNA in the ESR1-high group were significantly lower than in the group with clinically ER^+^ disease by IHC (Additional file 1: Figure S3).

The distribution of the PAM50 intrinsic subtypes within the TNBCtype subgroups was similar to previous reports where virtually all TNBCtype LAR tumors were non-Basal-like (i.e. HER2-enriched or luminal), and 42 % of MSL tumors were Normal-like (Additional file 1: Table S8 and Figure S4-5). Of note, 12.1 % of TNBCs subtyped by the TNBCtype (or 10.0 % of all TNBCs) were identified as UNS, and 86.0 % of these were of the Basal-like subtype by PAM50; thus, 27 % of the 350 clinically defined TNBCs were not assigned a biological group (i.e. either ESR1-high or UNS) by the TNBCtype tool (Fig. 2).

Of the remaining 290 TNBC sample set (350 TNBC – 60 removed for high ESR1), 271 patients with TNBC had response data (Additional file 1: Table S9). In this subset, the TNBCtype classification was not found to be significantly associated with pCR in univariate (*P* = 0.762) or multivariable analyses (*P* = 0.836). Of note, only eight patients had luminal A/B disease and their pCR rate was 25 % versus 41 % in non-luminal (Basal-like and HER2-enriched combined) tumors (OR = 0.477, 0.094–2.410).

Finally, we explored the ability of the TNBCtype classification to predict pCR within TNBC if the ESR1-high samples were included as an eighth subtype (i.e. ESR1-high). Interestingly, the pCR rate of the TNBCtype subtypes, as a single group, was significantly higher than the pCR rate of the ‘excluded’ TNBC ESR1-high group (39.9 % vs. 23.2 %, OR = 2.970, 1.221–7.222). In the entire TNBC population (n = 350), the TNBCtype classification that included the ESR1-high group was found significantly associated with pCR in multivariable analysis (*P* = 0.020) but not in univariate analysis (*P* = 0.239). When the TNBCtype + ESR1-high classification was included first in a multivariable model, addition of the PAM50 classification did not add independent predictive information, but was trending toward significance (*P* = 0.096). Similar results were obtained if the PAM50 classification was included first into the multivariable model and the TNBCtype + ESR1-high classification was added second (*P* = 0.088).

### Training and testing gene expression-based models predictive of pCR

We explored the ability of newly derived gene expression-based models to predict pCR in three different subgroups: all patients, patients with Basal-like disease, and patients with Luminal disease (Luminal A and B combined). To accomplish this, we built a model in the MDACC-based cohort (training dataset) and then tested the same model on the other cohorts (testing datasets) (Additional file 1: Figure S6-8).

In all patients, a gene expression-based model was identified in the MDACC-based cohort with an auROC of 0.80 (*P* <0.0001). This model predicted pCR in each testing datasets with auROC between 0.67-0.75 (*P* <0.001), and in the combined testing dataset (auROC 0.69, *P* <0.0001). The gene signatures that composed the model and whose high scores were associated with residual disease were correlation to the Luminal A centroid, correlation to PTEN present, and the Luminal A subtype (Additional file 1: Figure S6). Conversely, the gene signatures that composed the model and whose high scores were associated with pCR were correlation to the Basal-like centroid, correlation to PTEN absent [28], a beta-catenin signature, and a fetal mammary stem cell signature [29, 30].

In patients with Basal-like disease, a gene expression-based model was identified in the MDACC-based cohort (n = 166; auROC = 0.82, *P* <0.0001). This model predicted pCR in Horak et al. [19] (auROC 0.63, *P* = 0.018) and in the combined cohort of testing sets (n = 130; auROC 0.62, *P* = 0.011). Gene signatures that composed the model and whose high score were associated with residual disease were related to stromal/fibroblast-related biological processes (Additional file 1: Figure S7). Conversely, gene signatures that composed the model and whose high scores were associated with pCR were associated with histone/chromatin remodeling.

Finally, in patients with Luminal disease, a gene expression-based model was identified in the MDACC-based cohort (n = 254; auROC = 0.82, *P* <0.0001). This model predicted pCR in Miyake et al. [20] (auROC 0.76, *P* = 0.03) and in the combined cohort of testing sets (n = 195; auROC 0.64, *P* = 0.014). The only gene signature that composed the model and whose high score was associated with residual disease was correlation to TP53 wild-type status, whereas the only gene signature that composed the model and whose high score was associated with pCR was correlation to TP53 mutation (Additional file 1: Figure S8). Of note, both TP53 signatures composed our previously reported TP53 loss/mutation predictor [31].

## Discussion

Herein, we evaluated the association of the intrinsic subtypes of breast cancer with response and survival outcomes in a large combined dataset of newly diagnosed patients treated with multi-agent neoadjuvant chemotherapy and we made the following observations. First, the intrinsic subtypes of breast cancer provided independent prognostic information beyond standard clinical-pathological variables. Second, within patients that do not achieve a pCR, the ROR-P predictor can identify a group of patients with clinically node-negative disease with an excellent survival outcome at 5-years. Third, the intrinsic subtypes predict pCR and their predictive value is independent of standard clinicopathological variables. Fourth, the Basal-like subtype identifies a group of patients with a pCR rate >35 % across all pathology-based cohorts evaluated, including TNBC. Fifth, neither the identification of the Claudin-low subtype nor the recently reported seven-TNBC subtype classification predicted pCR within the large TNBC data set tested here, whereas the Luminal versus non-Luminal separation did predict pCR. Sixth, robust gene expression-based models predictive of pCR can be identified within all patients, Basal-like disease, and Luminal disease; however, additional validation of these new predictors is needed.

The intrinsic subtypes have previously been associated with outcome in patients that have not received adjuvant systemic therapy [32] and in patients that have received adjuvant endocrine therapy-only [33–38]. More recently, similar data has been observed in patients that have received adjuvant multi-agent chemotherapy, including CMF, anthracycline-based, and anthracycline/taxane-based chemotherapy regimens [5, 8, 33]. Concordant with the results of these studies, we observed an independent association of the intrinsic subtypes with DRFS in a population treated with cytotoxic and endocrine therapy (if HR^+^). Interestingly, this association with outcome was observed despite the fact that 20.3 % of the patients in the Hatzis et al. [19] dataset had an outstanding survival outcome at 5-years after achieving a pCR. This data reaffirms the strong prognostic ability of intrinsic subtyping in the context of standard adjuvant therapy.

The prognostic abilities of the PAM50 ROR-P have been clinically validated in two large retrospective cohorts from the ABCSG08 and transATAC phase III trials, where patients with surgically removed tumors received adjuvant endocrine therapy only [36, 37]. In this context, patients with a low ROR-P score have an outcome of distant metastasis-free survival at 10-years of 97.5 % [32], and these patients might be safely spared adjuvant (or neoadjuvant) chemotherapy. In our cohort of patients treated with neoadjuvant cytotoxic and adjuvant endocrine therapy (if HR^+^), ROR-P at diagnosis independently predicted DRFS and identified a low risk group of patients, especially within clinically node-negative disease, with an outstanding outcome (DRFS >95 % at 5-years). Similar results have been obtained with other prognostic signatures tested in patients with early breast cancer treated with and without multi-agent chemotherapy [39]. These nearly identical DRFS survival times with or without chemotherapy suggest that the potential survival benefit from neoadjuvant chemotherapy in patients with newly diagnosed breast cancer that is clinically node-negative and ROR-P low might be rather small, if any. In Hatzis et al. [19], the proportion of patients with ROR-P low within clinically node-negative disease was 26.8 %. If the main objective of neoadjuvant chemotherapy is to increase survival, then these patients with an outstanding baseline prognosis should be spared the toxic side-effects of chemotherapy and undergo surgical removal of their tumors.

Molecular classification of TNBC into subgroups that might be therapeutically relevant is an area of active and ongoing research. For example, the PAM50 assay identifies all the intrinsic molecular subtypes within TNBC, although Basal-like disease predominates [40]. In addition, we have identified and characterized a rare but relevant intrinsic subtype known as Claudin-low [4]. Interestingly, the intrinsic subtypes within TNBC share the same molecular features as the same subtypes within non-TNBC with the exception of the TNBC HER2-enriched tumors that do not show amplification of the ERBB2 17q amplicon [5, 41]. In our combined cohort of 350 TNBC cases, intrinsic subtyping, and especially the luminal versus non-luminal distinction, was found to be associated with pCR following neoadjuvant chemotherapy. However, the addition of the Claudin-low classification to the PAM50 classification did not improve these pCR versus no pCR predictions.

In addition, Lehmann et al. [17] have classified TNBC into seven subtypes (BL1, BL2, Immunomodulatory, LAR, Mesenchymal, MSL and UNS). This seven-subtype classification of TNBC has been found to be associated with pCR in an independent cohort of 143 patients with TNBC treated with anthracycline/taxane-based chemotherapy [18]. In our combined cohort of 290 TNBC cases with seven-subtype information, the Lehmann et al. [17] classification was not found to be significantly associated with pCR. However, concordant with a previous report, BL1 showed the highest pCR rate (i.e. 47 %) and BL2 the lowest pCR rate (i.e. 28 %). Surprisingly, the LAR group, which was found to have a 10 % (2/20) pCR rate in a previous report [18], showed a 37 % pCR rate in this larger combined cohort. This difference might be due to the fact that 71.4 % (20/28) of LAR tumors in our combined cohort were of the HER2-enriched subtype, a group of tumors highly responsive to chemotherapy, and only 17.9 % (5/28) were of the Luminal A/B subtype.

Two important issues of the Lehmann et al. [17] classification need to be taken into account. First, this classification ignores the Normal-like/normal tissue distinction. In other words, triple-negative tumors that are highly contaminated with normal breast tissue, which represent 11–16 % of the samples found in publicly available microarray datasets [17], are now classified into “tumor” subtypes. Whereas PAM50 identifies these tumors as being more similar to true normal breast samples (i.e. Normal-like) than to any tumor subtype, the Lehmann et al. [17] classification calls them as if they were a tumor (mostly MSL), although the Normal-like samples can also be observed in other subtype categories [40, 42]. Second, a substantial proportion of TNBC samples (~13–16 %) coming from the Lehmann et al. [17] classification were either not considered to be TNBC by gene expression and are removed (i.e. ESR1-high), or they fall into the unclassified or unstable (UNS) group, which is composed of a mix of tumors that only share the feature that they cannot be classified into one of the other six tumor subtypes.

This study also has other limitations that need to be highlighted. First, this was a retrospective and exploratory analysis of four datasets of patients treated with multi-agent chemotherapy; thus, we did not test a pre-specified hypothesis. Second, we used the research-based version of the PAM50 assay and not the standardized version that is currently commercially available. Third, we could not evaluate the predictive ability of the intrinsic subtypes to specific regimens or schedules. Fourth, we used the pathological data as provided in each publication and different definitions and cutoffs might have been used to determine the positivity of each biomarker. Thus, the results might have differed if ER, PR, and HER2 status had been centrally confirmed. Nonetheless, we and others have reported that, even within centrally confirmed TNBC, all the intrinsic molecular subtypes can be identified [15]. Fifth, Ki-67 by IHC was not available in any of the four datasets and thus we could not explore the ability of this biomarker to predict pCR following chemotherapy or survival outcome in the presence of the intrinsic subtypes or histological grade [43], especially within HR^+^/HER2^–^ disease. Sixth, the survival outcomes were only available in one of the datasets evaluated. Finally, the cutoffs to define the three risk groups of ROR-P were based on a large node-negative cohort that did not receive adjuvant systemic therapy [24]. These cutoffs might differ from the current standardized PAM50 version that takes into account tumor size and that defines the low risk group as those patients with a risk of distant relapse at 10-years below 3 % [36, 37].

## Conclusion

To conclude, intrinsic subtyping at diagnosis provides useful prognostic and predictive information for neoadjuvant chemotherapy-treated patients. The absolute benefit of chemotherapy in early breast cancer with clinically node-negative disease might be low if predicted to be ROR-P low risk at diagnosis. Further studies are needed to determine the role of intrinsic subtyping in treatment decision-making at diagnosis of breast cancer.

## Availability of data and materials

Four clinically annotated microarray-based breast cancer datasets were evaluated from the public domain (GSE25066 [19], GSE32646 [20], GSE41998 [21] and GSE22226 [22]). The sample names and subtype calls can be found in Additional file 2: Supplemental Data.

## Additional files

Additional file 1: Table S1. Cox model DRFS analyses including intrinsic subtype in all patients from the MDACC-based cohort (GSE25066). **Table S2.** Cox model DRFS analyses including ROR-P in all patients from the MDACC-based cohort (GSE25066). **Table S3.** Cox model DRFS analyses including intrinsic subtype in patients that achieved a pCR from the MDACC-based cohort (GSE25066). **Table S4.** Cox model DRFS analyses including ROR-P in patients that achieved a pCR from the MDACC-based cohort (GSE25066). **Table S5.** Cox model DRFS analyses including ROR-P in patients with residual disease from the MDACC-based cohort (GSE25066). **Table S6.** Distribution of the PAM50 subtypes within the TNBCtype groups and vice versa. **Table S7.** Association of the TNBCtype subtypes with chemotherapy response in triple-negative breast cancer. **Figure S1.** CONSORT diagram of the various cohorts evaluated in this study. **Figure S2.** Kaplan-Meier distant relapse-free survival analysis in MDACC-based (GSE25066 [13]) dataset set. (A) Survival outcomes of the ROR-P groups in all patients. (B) Survival outcomes of the ROR-P groups in patients with clinically node-negative disease. **Figure S3.** Levels of ESR1 across TNBCtype ESR1-low group, TNBCtype ESR1-high group and ER^+^ group. Median expression of ESR1 in the PAM50 training dataset reported in Parker et al. [24] has been set to zero. **Figure S4.** Distribution of the TNBCtype subtypes and ESR1-high group within the PAM50 subtypes in TNBC. **Figure S5.** Distribution of the TNBCtype subtypes and ESR1-high group within the PAM50 + Claudin-low subtypes in TNBC. **Figure S6.** Training and testing gene expression-based models predictive of pCR in all patients. **Figure S7.** Training and testing gene expression-based models predictive of pCR in patients with Basal-like disease. **Figure S8.** Training and testing gene expression-based models predictive of pCR in patients with luminal (A/B) disease. (DOCX 819 kb) Additional file 2:
**Supplemental data.** (XLSX 897 kb)

## Abbreviations

auROC: Area under the receiver operating characteristic
BL1: Basal 1
BL2: Basal 2
DRFS: Distant relapse-free survival
ER: Estrogen receptor
ESR1: ER gene
IHC: Immunohistochemistry
LAR: Luminal androgen receptor
MSL: Mesenchymal stem cell
pCR: Pathological complete response
PR: Progesterone receptor
ROR-P: Risk of relapse score based on subtype and proliferation
TNBC: Triple-negative breast cancer
UNS: Unstable
