# Supplementary material for: Response and survival of breast cancer intrinsic subtypes following multi-agent neoadjuvant chemotherapy
Source: BMC Med. 2015 Dec 18;13:303. doi: 10.1186/s12916-015-0540-z (PMC4683815; doi:10.1186/s12916-015-0540-z)
Supplement: Additional file 1: Table S1. — Cox model DRFS analyses including intrinsic subtype in all patients from the MDACC-based cohort (GSE25066). Table S2. Cox model DRFS analyses including ROR-P in all patients from the MDACC-based cohort (GSE25066). Table S3. Cox model DRFS analyses including intrinsic subtype in patients that achieved a pCR from the MDACC-based cohort (GSE25066). Table S4. Cox model DRFS analyses including ROR-P in patients that achieved a pCR from the MDACC-based cohort (GSE25066). Table S5. Cox model DRFS analyses including ROR-P in patients with residual disease from the MDACC-based cohort (GSE25066). Table S6. Distribution of the PAM50 subtypes within the TNBCtype groups and vice versa. Table S7. Association of the TNBCtype subtypes with chemotherapy response in triple-negative breast cancer. Figure S1. CONSORT diagram of the various cohorts evaluated in this study. Figure S2. Kaplan-Meier distant relapse-free survival analysis in MDACC-based (GSE25066 [13]) dataset set. (A) Survival outcomes of the ROR-P groups in all patients. (B) Survival outcomes of the ROR-P groups in patients with clinically node-negative disease. Figure S3. Levels of ESR1 across TNBCtype ESR1-low group, TNBCtype ESR1-high group and ER+ group. Median expression of ESR1 in the PAM50 training dataset reported in Parker et al. [24] has been set to zero. Figure S4. Distribution of the TNBCtype subtypes and ESR1-high group within the PAM50 subtypes in TNBC. Figure S5. Distribution of the TNBCtype subtypes and ESR1-high group within the PAM50 + Claudin-low subtypes in TNBC. Figure S6. Training and testing gene expression-based models predictive of pCR in all patients. Figure S7. Training and testing gene expression-based models predictive of pCR in patients with Basal-like disease. Figure S8. Training and testing gene expression-based models predictive of pCR in patients with luminal (A/B) disease. (DOCX 819 kb) [file 12916_2015_540_MOESM1_ESM.docx]

***Online Only Tables and Figure Legends***

*Response and Survival of the Breast Cancer Intrinsic Subtypes following Multi-Agent Neoadjuvant Chemotherapy*

**Tables**

**Table S1**. Cox model DRFS analyses including intrinsic subtype in all patients from the MDACC-based cohort (GSE25066).

**Table S2**. Cox model DRFS analyses including ROR-P in all patients from the MDACC-based cohort (GSE25066).

**Table S3**. Cox model DRFS analyses including intrinsic subtype in patients that achieved a pCR from the MDACC-based cohort (GSE25066).

**Table S4**. Cox model DRFS analyses including ROR-P in patients that achieved a pCR from the MDACC-based cohort (GSE25066).

**Table S5**. Cox model DRFS analyses including ROR-P in patients with residual disease from the MDACC-based cohort (GSE25066).

**Table S6**. Distribution of the PAM50 subtypes within the TNBCtype groups and viceversa.

**Table S7.** Association of the TNBCtype subtypes with chemotherapy response in triple-negative breast cancer.

**Figures**

**Figure S1**. CONSORT diagram of the various cohorts evaluated in this study.

**Figure S2**. Kaplan-Meier distant relapse-free survival analysis in MDACC-based (GSE25066[^13^](#_ENREF_13)) dataset set. (**A**) Survival outcomes of the ROR-P groups in all patients. (**B**) Survival outcomes of the ROR-P groups in patients with clinically node-negative disease.

**Figure S3**. Levels of ESR1 across TNBCtype ESR1-low group, TNBCtype ESR1-high group and ER-positive group. Median expression of ESR1 in the PAM50 training dataset reported in Parker et al. has been set to zero.

**Figure S4**. Distribution of the TNBCtype subtypes and ESR1-high group within the PAM50 subtypes in TNBC.

**Figure S5**. Distribution of the TNBCtype subtypes and ESR1-high group within the PAM50+Claudin-low subtypes in TNBC.

**Figure S6**. Training and testing gene expression-based models predictive of pCR in all patients.

**Figure S7**. Training and testing gene expression-based models predictive of pCR in patients with Basal-like disease.

**Figure S8**. Training and testing gene expression-based models predictive of pCR in patients with luminal (A+B) disease.

**Table S1**. Cox model DRFS analyses including intrinsic subtype in all patients from the MDACC-based cohort (GSE25066).

|  |  |  |  |  | **Univariate Analysis** | | | |  | **Multivariable Analysis** | | | |
| --- | --- | --- | --- | --- | --- | --- | --- | --- | --- | --- | --- | --- | --- |
|  |  |  |  |  |  |  |  |  |  |  |  |  |  |
| **Variables** | **N** | **%** | **5-yr DRFS** |  | **HR** | **Lower 95%** | **Upper 95%** | **p-value** |  | **HR** | **Lower 95%** | **Upper 95%** | **p-value** |
| Age (cont. variable) | - | - | - |  | 1.0 | 0.98 | 1.02 | 0.860 |  | 0.98 | 0.97 | 1.00 | 0.120 |
| Tumor size |  |  |  |  |  |  |  |  |  |  |  |  |  |
| T0-T2 | 288 | 57% | 76% |  | 1.0 | - | - | - |  | 1.0 | - | - | - |
| T3-T4 | 220 | 43% | 68% |  | 1.8 | 1.23 | 2.60 | 0.002 |  | 1.4 | 1.12 | 1.84 | 0.004 |
| Node status |  |  |  |  |  |  |  |  |  |  |  |  |  |
| N0 | 157 | 31% | 86% |  | 1.0 | - | - | - |  | 1.0 | - | - | - |
| N1-3 | 351 | 69% | 66% |  | 2.9 | 1.74 | 4.91 | <0.001 |  | 3.1 | 1.70 | 5.59 | <0.001 |
| ER IHC |  |  |  |  |  |  |  |  |  |  |  |  |  |
| Positive | 297 | 59% | 80% |  | 1.0 | - | - | - |  | 1.0 | - | - | - |
| Negative | 205 | 41% | 61% |  | 2.9 | 1.97 | 4.27 | <0.001 |  | 1.7 | 0.87 | 3.46 | 0.110 |
| PR IHC |  |  |  |  |  |  |  |  |  |  |  |  |  |
| Positive | 243 | 49% | 81% |  | 1.0 | - | - | - |  | 1.0 | - | - | - |
| Negative | 258 | 51% | 64% |  | 2.6 | 1.75 | 3.96 | <0.001 |  | 1.2 | 0.68 | 2.25 | 0.490 |
| HER2 STATUS |  |  |  |  |  |  |  |  |  |  |  |  |  |
| Negative | 485 | 99% | 72% |  | 1.0 | - | - | - |  | 1.0 | - | - | - |
| Positive | 6 | 1% | 67% |  | 1.8 | 0.43 | 7.13 | 0.430 |  | 0.5 | 0.06 | 3.40 | 0.440 |
| Histological Grade |  |  |  |  |  |  |  |  |  |  |  |  |  |
| 1 | 32 | 7% | 97% |  | 1.0 | - | - | - |  | 1.0 | - | - | - |
| 2 | 180 | 38% | 74% |  | 7.0 | 0.96 | 51.23 | 0.055 |  | 2.79 | 0.37 | 21.22 | 0.32 |
| 3 | 259 | 55% | 69% |  | 9.5 | 1.31 | 68.25 | 0.026 |  | 2.58 | 0.33 | 20.01 | 0.36 |
| PAM50 |  |  |  |  |  |  |  |  |  |  |  |  |  |
| Luminal A | 155 | 33% | 81% |  | 1.0 | - | - | - |  | 1.0 | - | - | - |
| Luminal B | 109 | 23% | 78% |  | 1.4 | 0.71 | 2.64 | 0.344 |  | 1.4 | 0.67 | 2.76 | 0.390 |
| HER2-E | 40 | 8% | 58% |  | 3.4 | 1.69 | 6.75 | 0.001 |  | 2.7 | 1.15 | 6.24 | 0.022 |
| Basal-like | 171 | 36% | 63% |  | 3.5 | 2.09 | 5.94 | <0.001 |  | 2.6 | 1.11 | 5.98 | 0.027 |
| pCR |  |  |  |  |  |  |  |  |  |  |  |  |  |
| No | 389 | 80% | 92% |  | 1.0 | - | - | - |  | 1.0 | - | - | - |
| Yes | 99 | 20% | 68% |  | 0.3 | 0.12 | 0.55 | 0.001 |  | 0.11 | 0.04 | 0.27 | <0.001 |

**Table S2**. Cox model DRFS analyses including ROR-P in all patients from the MDACC-based cohort (GSE25066).

|  |  |  |  |  | **Univariable Analysis** | | | |  | **Multivariable Analysis** | | | |
| --- | --- | --- | --- | --- | --- | --- | --- | --- | --- | --- | --- | --- | --- |
|  |  |  |  |  |  |  |  |  |  |  |  |  |  |
| **Variables** | **N** | **%** | **5-yr DRFS** |  | **HR** | **Lower 95%** | **Upper 95%** | **p-value** |  | **HR** | **Lower 95%** | **Upper 95%** | **p-value** |
| Age (cont. variable) | - | - | - |  | 1.0 | 0.98 | 1.02 | 0.860 |  | 1.00 | 0.97 | 1.01 | 0.170 |
| Tumor size |  |  |  |  |  |  |  |  |  |  |  |  |  |
| T0-T2 | 288 | 57% | 76% |  | 1.0 | - | - | - |  | 1.0 | - | - | - |
| T3-T4 | 220 | 43% | 68% |  | 1.8 | 1.23 | 2.60 | 0.002 |  | 1.3 | 1.03 | 1.65 | 0.030 |
| Node status |  |  |  |  |  |  |  |  |  |  |  |  |  |
| N0 | 157 | 31% | 86% |  | 1.0 | - | - | - |  | 1.0 | - | - | - |
| N1-3 | 351 | 69% | 66% |  | 2.9 | 1.74 | 4.91 | <0.001 |  | 2.9 | 1.62 | 5.11 | <0.001 |
| ER IHC |  |  |  |  |  |  |  |  |  |  |  |  |  |
| Positive | 297 | 59% | 80% |  | 1.0 | - | - | - |  | 1.0 | - | - | - |
| Negative | 205 | 41% | 61% |  | 2.9 | 1.97 | 4.27 | <0.001 |  | 2.6 | 1.47 | 4.49 | 0.001 |
| PR IHC |  |  |  |  |  |  |  |  |  |  |  |  |  |
| Positive | 243 | 49% | 81% |  | 1.0 | - | - | - |  | 1.0 | - | - | - |
| Negative | 258 | 51% | 64% |  | 2.6 | 1.75 | 3.96 | <0.001 |  | 1.3 | 0.73 | 2.28 | 0.372 |
| HER2 STATUS |  |  |  |  |  |  |  |  |  |  |  |  |  |
| Negative | 485 | 99% | 72% |  | 1.0 | - | - | - |  | 1.0 | - | - | - |
| Positive | 6 | 1% | 67% |  | 1.8 | 0.43 | 7.13 | 0.430 |  | 0.4 | 0.06 | 3.18 | 0.406 |
| Histological Grade |  |  |  |  |  |  |  |  |  |  |  |  |  |
| 1 | 32 | 7% | 97% |  | 1.0 | - | - | - |  | 1.0 | - | - | - |
| 2 | 180 | 38% | 74% |  | 7.0 | 0.96 | 51.23 | 0.055 |  | 3.27 | 0.44 | 24.45 | 0.25 |
| 3 | 259 | 55% | 69% |  | 9.5 | 1.31 | 68.25 | 0.026 |  | 2.74 | 0.37 | 20.55 | 0.33 |
| ROR-P |  |  |  |  |  |  |  |  |  |  |  |  |  |
| Low | 80 | 21% | 92% |  | 1.0 | - | - | - |  | 1.0 | - | - | - |
| Med | 206 | 53% | 68% |  | 4.8 | 1.92 | 12.06 | <0.001 |  | 2.8 | 1.07 | 7.15 | 0.037 |
| High | 103 | 26% | 48% |  | 10.0 | 3.95 | 25.15 | <0.001 |  | 4.4 | 1.65 | 11.66 | 0.003 |
| pCR |  |  |  |  |  |  |  |  |  |  |  |  |  |
| No | 389 | 80% | 92% |  | 1.0 | - | - | - |  | 1.0 | - | - | - |
| Yes | 99 | 20% | 68% |  | 0.3 | 0.12 | 0.55 | 0.001 |  | 0.1 | 0.05 | 0.28 | <0.001 |

**Table S3**. Cox model DRFS analyses including intrinsic subtype in patients that achieved a pCR from the MDACC-based cohort (GSE25066).

|  |  |  |  |  | **Univariable Analysis** | | | |
| --- | --- | --- | --- | --- | --- | --- | --- | --- |
|  |  |  |  |  |  |  |  |  |
| **Variables** | **N** | **%** | **5-yr DRFS** |  | **HR** | **Lower 95%** | **Upper 95%** | **p-value** |
| Age (cont. variable) | - | - | - |  | 1.0 | 0.91 | 1.08 | 0.880 |
| Tumor size |  |  |  |  |  |  |  |  |
| T0-T2 | 58 | 59% | 90% |  | 1.0 | - | - | - |
| T3-T4 | 41 | 41% | 95% |  | 0.6 | 0.11 | 2.88 | 0.486 |
| Node status |  |  |  |  |  |  |  |  |
| N0 | 28 | 28% | 96% |  | 1.0 | - | - | - |
| N1-3 | 71 | 72% | 91% |  | 2.5 | 0.30 | 20.49 | 0.403 |
| ER IHC |  |  |  |  |  |  |  |  |
| Positive | 30 | 31% | 100% |  | 1.0 | - | - | - |
| Negative | 68 | 69% | 90% |  | - | 0.00 | Inf | 0.999 |
| PR IHC |  |  |  |  |  |  |  |  |
| Positive | 26 | 27% | 100% |  | 1.0 | - | - | - |
| Negative | 72 | 73% | 90% |  | - | 0.00 | Inf | 0.999 |
| HER2 STATUS |  |  |  |  |  |  |  |  |
| Negative | 93 | 98% | 93% |  | 1.0 | - | - | - |
| Positive | 2 | 2% | 100% |  | - | 0.00 | Inf | 0.999 |
| Histological Grade |  |  |  |  |  |  |  |  |
| 1 | 1 | 1% | 100% |  | 1.0 | - | - | - |
| 2 | 12 | 13% | 100% |  | - | 0.00 | Inf | 0.999 |
| 3 | 77 | 86% | 92% |  | - | 0.00 | Inf | 0.999 |
| PAM50 |  |  |  |  |  |  |  |  |
| Luminal A | 4 | 4% | 100% |  | 1.0 | - | - | - |
| Luminal B | 16 | 17% | 100% |  | - | 0.00 | Inf | 0.999 |
| HER2-E | 9 | 10% | 100% |  | - | 0.00 | Inf | 0.999 |
| Basal-like | 64 | 69% | 88% |  | - | 0.00 | Inf | 0.999 |

**Table S4**. Cox model DRFS analyses including ROR-P in patients that achieved a pCR from the MDACC-based cohort (GSE25066).

|  |  |  |  |  | **Univariable Analysis** | | | |
| --- | --- | --- | --- | --- | --- | --- | --- | --- |
|  |  |  |  |  |  |  |  |  |
| **Variables** | **N** | **%** | **5-yr DRFS** |  | **HR** | **Lower 95%** | **Upper 95%** | **p-value** |
| Age (cont. variable) | - | - | - |  | 1.0 | 0.91 | 1.08 | 0.880 |
| Tumor size |  |  |  |  |  |  |  |  |
| T0-T2 | 58 | 59% | 90% |  | 1.0 | - | - | - |
| T3-T4 | 41 | 41% | 95% |  | 0.6 | 0.11 | 2.88 | 0.486 |
| Node status |  |  |  |  |  |  |  |  |
| N0 | 28 | 28% | 96% |  | 1.0 | - | - | - |
| N1-3 | 71 | 72% | 91% |  | 2.5 | 0.30 | 20.49 | 0.403 |
| ER IHC |  |  |  |  |  |  |  |  |
| Positive | 30 | 31% | 100% |  | 1.0 | - | - | - |
| Negative | 68 | 69% | 90% |  | - | 0.00 | Inf | 0.999 |
| PR IHC |  |  |  |  |  |  |  |  |
| Positive | 26 | 27% | 100% |  | 1.0 | - | - | - |
| Negative | 72 | 73% | 90% |  | - | 0.00 | Inf | 0.999 |
| HER2 STATUS |  |  |  |  |  |  |  |  |
| Negative | 93 | 98% | 93% |  | 1.0 | - | - | - |
| Positive | 2 | 2% | 100% |  | - | 0.00 | Inf | 0.999 |
| Histological Grade |  |  |  |  |  |  |  |  |
| 1 | 1 | 1% | 100% |  | 1.0 | - | - | - |
| 2 | 12 | 13% | 100% |  | - | 0.00 | Inf | 0.999 |
| 3 | 77 | 86% | 92% |  | - | 0.00 | Inf | 0.999 |
| ROR-P |  |  |  |  |  |  |  |  |
| Low | 6 | 6% | - |  | 1.0 | - | - | - |
| Med | 50 | 51% | 91% |  | - | 0.00 | Inf | 0.999 |
| High | 43 | 43% | 92% |  | - | 0.00 | Inf | 0.999 |

**Table S5**. Cox model DRFS analyses including ROR-P in patients with residual disease from the MDACC-based cohort (GSE25066).

|  |  |  |  |  | **Univariable Analysis** | | | |  | **Multivariable Analysis** | | | |
| --- | --- | --- | --- | --- | --- | --- | --- | --- | --- | --- | --- | --- | --- |
|  |  |  |  |  |  |  |  |  |  |  |  |  |  |
| **Variables** | **N** | **%** | **5-yr DRFS** |  | **HR** | **Lower 95%** | **Upper 95%** | **p-value** |  | **HR** | **Lower 95%** | **Upper 95%** | **p-value** |
| Age (cont. variable) | - | - | - |  | 1.0 | 0.98 | 1.01 | 0.590 |  | 0.99 | 0.97 | 1.01 | 0.186 |
| Tumor size |  |  |  |  |  |  |  |  |  |  |  |  |  |
| T0-T2 | 216 | 56% | 74% |  | 1.0 | - | - | - |  | 1.0 | - | - | - |
| T3-T4 | 173 | 44% | 61% |  | 2.1 | 1.38 | 3.07 | <0.001 |  | 1.3 | 1.05 | 1.71 | 0.018 |
| Node status |  |  |  |  |  |  |  |  |  |  |  |  |  |
| N0 | 126 | 32% | 85% |  | 1.0 | - | - | - |  | 1.0 | - | - | - |
| N1-3 | 263 | 68% | 66% |  | 3.3 | 1.90 | 5.71 | <0.001 |  | 2.7 | 1.54 | 4.90 | <0.001 |
| ER IHC |  |  |  |  |  |  |  |  |  |  |  |  |  |
| Positive | 129 | 34% | 78% |  | 1.0 | - | - | - |  | 1.0 | - | - | - |
| Negative | 255 | 66% | 47% |  | 4.0 | 2.69 | 6.02 | <0.001 |  | 2.6 | 1.47 | 4.61 | 0.001 |
| PR IHC |  |  |  |  |  |  |  |  |  |  |  |  |  |
| Positive | 175 | 46% | 79% |  | 1.0 | - | - | - |  | 1.0 | - | - | - |
| Negative | 208 | 54% | 54% |  | 3.3 | 2.15 | 5.05 | <0.001 |  | 1.2 | 0.67 | 2.15 | 0.539 |
| HER2 STATUS |  |  |  |  |  |  |  |  |  |  |  |  |  |
| Negative | 373 | 99% | 68% |  | 1.0 | - | - | - |  | 1.0 | - | - | - |
| Positive | 3 | 1% | NA |  | 1.1 | 0.16 | 8.16 | 0.900 |  | 0.4 | 0.06 | 3.26 | 0.419 |
| Histological Grade |  |  |  |  |  |  |  |  |  |  |  |  |  |
| 1 | 28 | 8% | 96% |  | 1.0 | - | - | - |  | 1.0 | - | - | - |
| 2 | 160 | 44% | 73% |  | 6.1 | 0.83 | 44.43 | 0.076 |  | 3.26 | 0.43 | 24.43 | 0.25 |
| 3 | 175 | 48% | 60% |  | 10.9 | 1.51 | 78.73 | 0.018 |  | 2.70 | 0.36 | 20.29 | 0.33 |
| ROR-P |  |  |  |  |  |  |  |  |  |  |  |  |  |
| Low | 80 | 21% | 92% |  | 1.0 | - | - | - |  | 1.0 | - | - | - |
| Med | 206 | 53% | 68% |  | 4.8 | 1.92 | 12.06 | <0.001 |  | 2.7 | 1.05 | 7.10 | 0.039 |
| High | 103 | 26% | 48% |  | 10.0 | 3.95 | 25.15 | <0.001 |  | 4.6 | 1.71 | 12.22 | 0.003 |

**Table S6.** Logistic regression model analyses of chemotherapy response in the combined cohort (except Horak et al.).

|  |  |  |  | **Univariate Analysis** | | | | |  | **Multivariate Analysis** | | | | |
| --- | --- | --- | --- | --- | --- | --- | --- | --- | --- | --- | --- | --- | --- | --- |
|  |  |  |  |  |  |  |  |  |  |  |  |  |  |  |
| **Signatures** | **N** | **pCR rate** |  | **OR** | **Lower 95%** | **Upper 95%** | **p-value** | **aROC** |  | **OR** | **Lower 95%** | **Upper 95%** | **p-value** | **aROC** |
| Age (cont. variable) | - | - |  | 1.0 | 0.97 | 1.01 | 0.375 | 0.521 |  | 1.00 | 0.97 | 1.02 | 0.749 | 0.786 |
| Tumor size |  |  |  |  |  |  |  |  |  |  |  |  |  |  |
| T0-T2 | 392 | 23% |  | 1.0 | - | - | - | 0.529 |  | 1.0 | - | - | - |  |
| T3-T4 | 264 | 19% |  | 0.8 | 0.53 | 1.15 | 0.207 |  |  | 0.6 | 0.50 | 0.30 | 0.807 |  |
| ER IHC |  |  |  |  |  |  |  |  |  |  |  |  |  |  |
| Positive | 386 | 12% |  | 1.0 | - | - | - | 0.678 |  | 1.0 | - | - | - |  |
| Negative | 263 | 37% |  | 4.4 | 2.97 | 6.60 | <0.001 |  |  | 1.3 | 0.61 | 2.58 | 0.533 |  |
| PR IHC |  |  |  |  |  |  |  |  |  |  |  |  |  |  |
| Positive | 299 | 12% |  | 1.0 | - | - | - | 0.638 |  | 1.0 | - | - | - |  |
| Negative | 349 | 31% |  | 3.3 | 2.19 | 5.07 | <0.001 |  |  | 0.8 | 0.40 | 1.51 | 0.463 |  |
| HER2 STATUS |  |  |  |  |  |  |  |  |  |  |  |  |  |  |
| Negative | 570 | 20% |  | 1.0 | - | - | - | 0.551 |  | 1.0 | - | - | - |  |
| Positive | 64 | 39% |  | 2.6 | 1.51 | 4.46 | 0.001 |  |  | 2.1 | 0.90 | 4.88 | 0.087 |  |
| GRADE |  |  |  |  |  |  |  |  |  |  |  |  |  |  |
| Grade 1 | 46 | 7% |  | 1.0 | - | - | - | 0.656 |  | 1.0 | - | - | - |  |
| Grade 2 | 277 | 13% |  | 2.1 | 0.61 | 7.04 | 0.243 |  |  | 0.7 | 0.17 | 2.85 | 0.617 |  |
| Grade 3 | 299 | 32% |  | 6.8 | 2.05 | 22.40 | 0.002 |  |  | 1.5 | 0.35 | 6.50 | 0.578 |  |
| PAM50 |  |  |  |  |  |  |  |  |  |  |  |  |  |  |
| Luminal A | 195 | 4% |  | 1.0 | - | - | - | 0.736 |  | 1.0 | - | - | - |  |
| Luminal B | 138 | 16% |  | 5.1 | 2.11 | 12.30 | <0.001 |  |  | 4.5 | 1.71 | 11.75 | 0.002 |  |
| HER2-E | 74 | 34% |  | 13.7 | 5.60 | 33.54 | <0.001 |  |  | 8.3 | 2.74 | 24.85 | <0.001 |  |
| Basal-like | 198 | 39% |  | 17.1 | 7.63 | 38.29 | <0.001 |  |  | 13.2 | 4.50 | 38.50 | <0.001 |  |
| Normal-like | 52 | 23% |  | 8.1 | 2.99 | 21.74 | <0.001 |  |  | - | - | - | - |  |
| STUDY |  |  |  |  |  |  |  |  |  |  |  |  |  |  |
| MDACC508 | 488 | 20% |  | 1.0 | - | - | - | 0.536 |  | 1.0 | - | - | - |  |
| ISPY | 54 | 31% |  | 1.8 | 0.98 | 3.34 | 0.060 |  |  | 1.7 | 0.63 | 4.37 | 0.301 |  |
| MIYAKE | 115 | 23% |  | 1.2 | 0.74 | 1.96 | 0.449 |  |  | 1.5 | 0.75 | 3.12 | 0.238 |  |

*OR, odds ratio; aROC, area under the receiver operating curve.

**Table S7**. Pathological complete response (pCR) rates of the PAM50+Claudin-low subtype classification*. (**A**) Within patients with triple-negative disease and (**B**) within all patients.

**A**

|  | **RD** | **%** | **pCR** | **%** | **Total** |
| --- | --- | --- | --- | --- | --- |
| Luminal A | 9 | 75.0% | 3 | 25.0% | 12 |
| Luminal B | 7 | 100.0% | 0 | 0.0% | 7 |
| Basal-like | 119 | 64.3% | 66 | 35.7% | 185 |
| HER2-E | 16 | 57.1% | 12 | 42.9% | 28 |
| Normal-like | 12 | 60.0% | 8 | 40.0% | 20 |
| Claudin-low | 43 | 57.3% | 32 | 42.7% | 75 |

**B**

|  | **RD** | **%** | **pCR** | **%** | **Total** |
| --- | --- | --- | --- | --- | --- |
| Luminal A | 248 | 94.7% | 14 | 5.3% | 262 |
| Luminal B | 140 | 84.3% | 26 | 15.7% | 166 |
| Basal-like | 148 | 62.4% | 89 | 37.6% | 237 |
| HER2-E | 56 | 65.1% | 30 | 34.9% | 86 |
| Normal-like | 35 | 71.4% | 14 | 28.6% | 49 |
| Claudin-low | 71 | 64.5% | 39 | 35.5% | 110 |

*RD, residual disease.


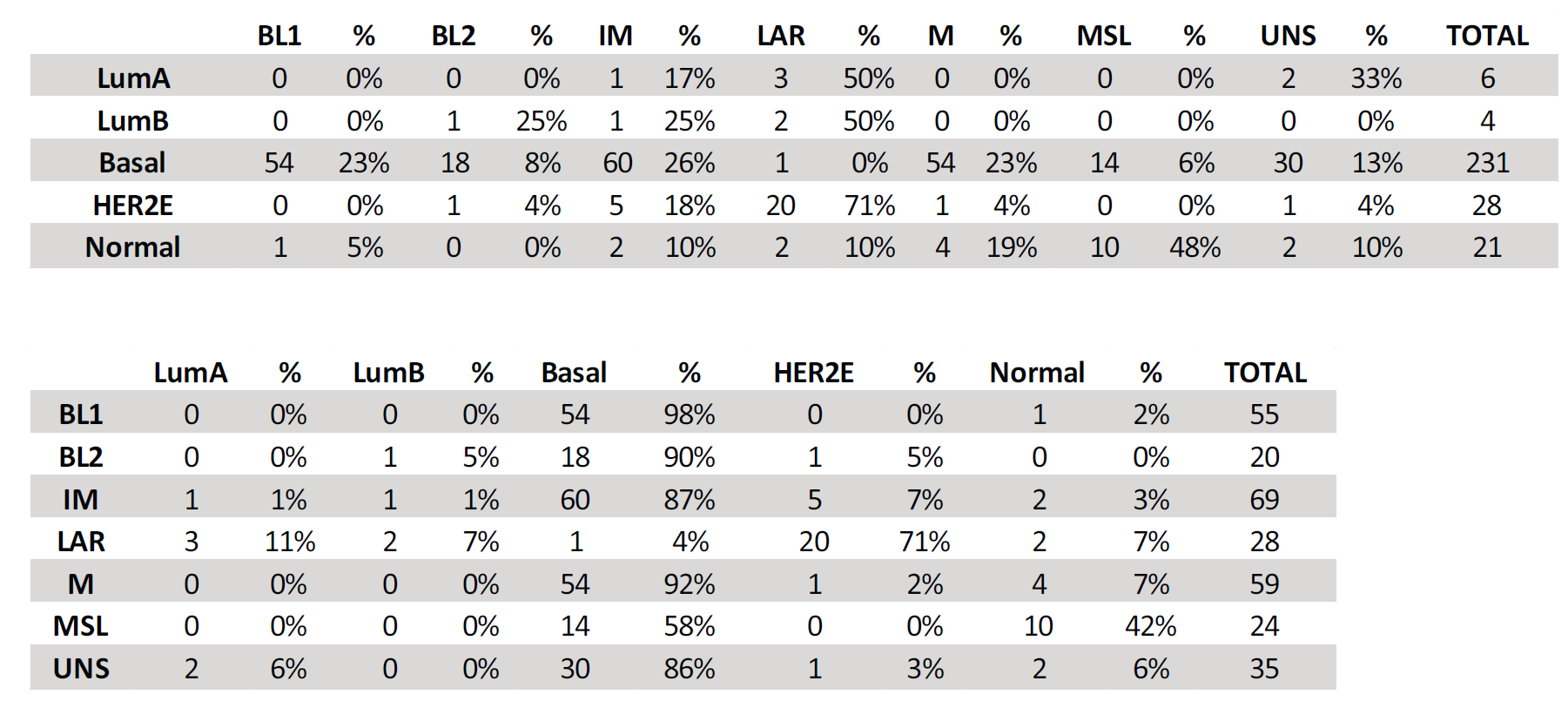
**Table S8**. Distribution of the PAM50 subtypes within the TNBCtype groups and vice versa*.

*HER2E, HER2-enriched; BL1, Basal 1; BL2, Basal 2; IM, immunomodulatory; LAR, luminal androgen receptor; M, mesenchymal; MSL, mesenchymal stem cell; UNS, unstable.

**Table S9.** Association of the TNBCtype subtypes with chemotherapy response in triple-negative breast cancer.

|  | **HR-/HER2- (TN)** | | | | | | |  |  |
| --- | --- | --- | --- | --- | --- | --- | --- | --- | --- |
|  | **N** | **%** |  | **pCR** | **%** | **RD** | **%** |  | **Adjusted P-value*** |
| **BL1** | 54 | 20% |  | 25 | 46% | 29 | 54% |  | 0.140 |
| **BL2** | 18 | 7% |  | 5 | 28% | 13 | 72% |  |  |
| **IM** | 67 | 25% |  | 25 | 37% | 42 | 63% |  |  |
| **M** | 50 | 18% |  | 18 | 36% | 32 | 64% |  |  |
| **LAR** | 27 | 10% |  | 10 | 37% | 17 | 63% |  |  |
| **MSL** | 22 | 8% |  | 10 | 45% | 12 | 55% |  |  |
| **UNS** | 33 | 12% |  | 15 | 45% | 18 | 55% |  |  |

*, Likelihood ratio test: adjusting clinical features: age, clinical stage, clinical nodal status and study cohort. In the model, TNBCtype subtype was used as categorical variable.

**Figure S1**


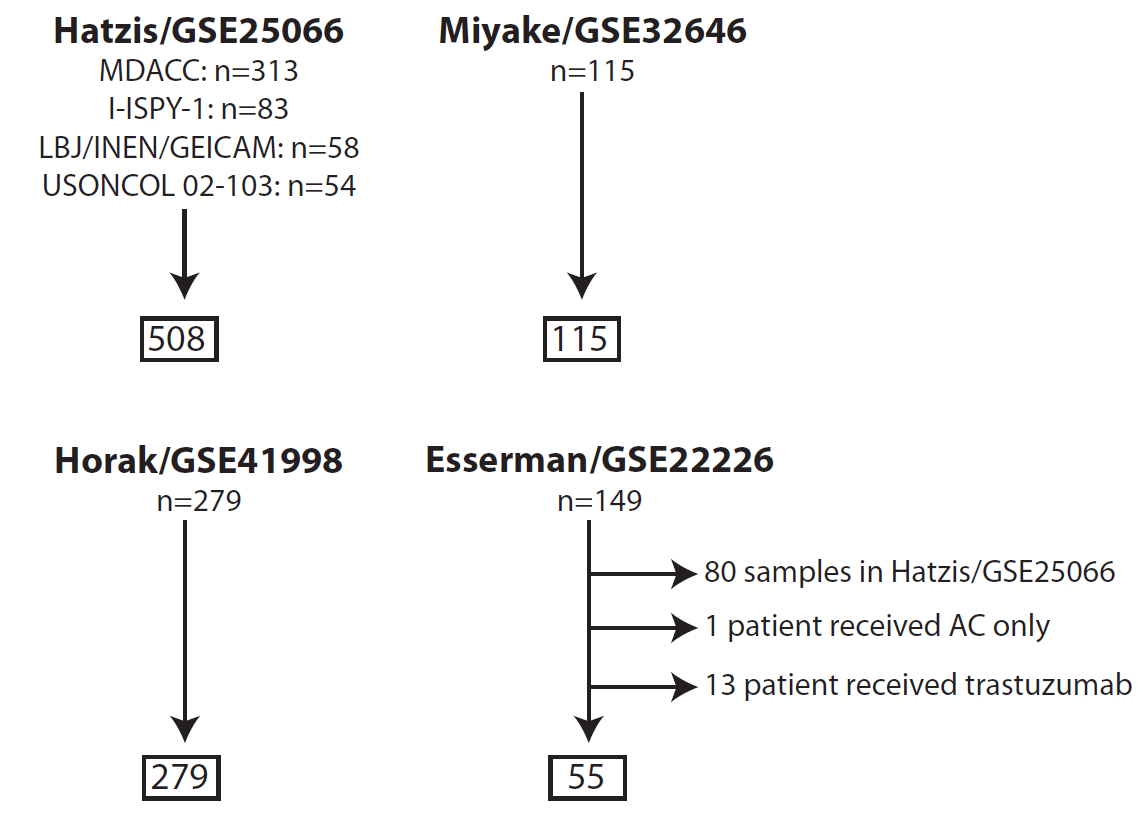

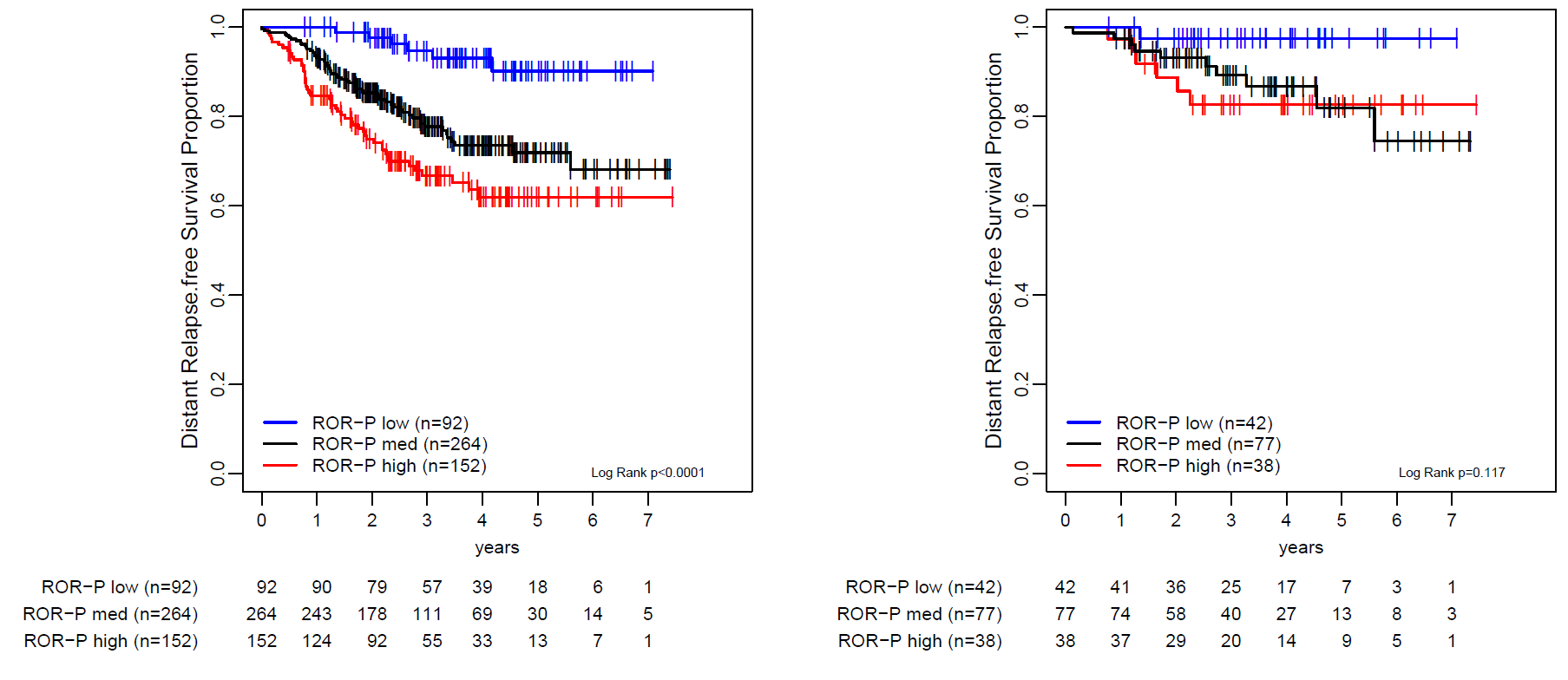
**Figure S2**

**Figure S3**


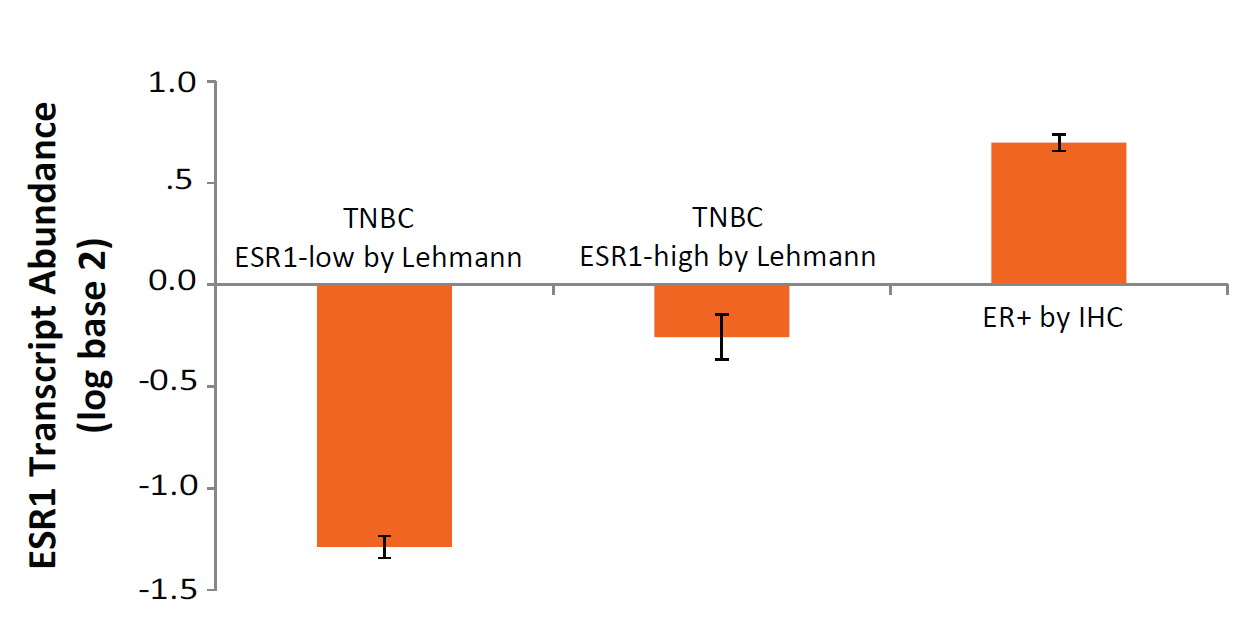


**Figure S4**


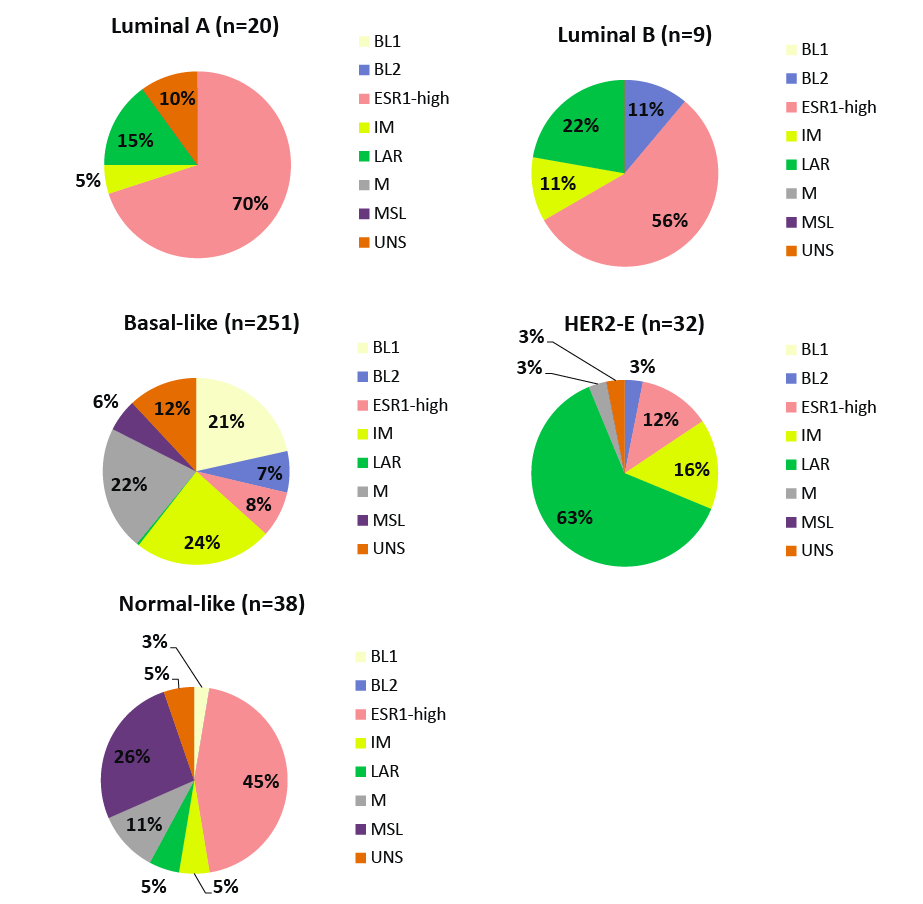


**Figure S5**


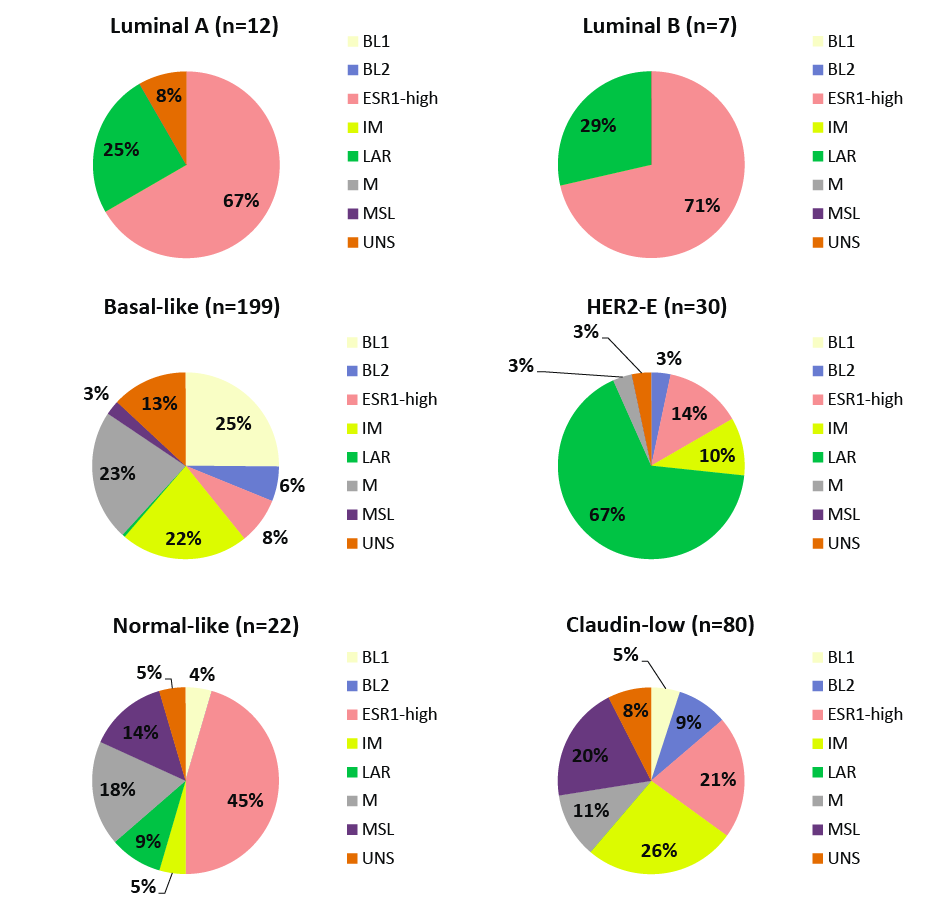


**Figure S6**


**Figure S7**

**Figure S8**
